# Redox-Promoted Tailoring of the High-Temperature Electrical Performance in Ca_3_Co_4_O_9_ Thermoelectric Materials by Metallic Cobalt Addition

**DOI:** 10.3390/ma13051060

**Published:** 2020-02-27

**Authors:** Gabriel Constantinescu, Artur R. Sarabando, Shahed Rasekh, Diogo Lopes, Sergii Sergiienko, Parisa Amirkhizi, Jorge R. Frade, Andrei V. Kovalevsky

**Affiliations:** Department of Materials and Ceramic Engineering, CICECO–Aveiro Institute of Materials, University of Aveiro, 3810-193 Aveiro, Portugal; artursarabando@ua.pt (A.R.S.); shahedvrm@ua.pt (S.R.); djlopes@ua.pt (D.L.); sergiienko@ua.pt (S.S.); parisa.amirkhizi@ua.pt (P.A.); jfrade@ua.pt (J.R.F.); akavaleuski@ua.pt (A.V.K.)

**Keywords:** calcium cobaltite, TE performance, electrical properties, composite, redox tuning

## Abstract

This paper reports a novel composite-based processing route for improving the electrical performance of Ca_3_Co_4_O_9_ thermoelectric (TE) ceramics. The approach involves the addition of metallic Co, acting as a pore filler on oxidation, and considers two simple sintering schemes. The (1-x)Ca_3_Co_4_O_9_/xCo composites (x = 0%, 3%, 6% and 9% vol.) have been prepared through a modified Pechini method, followed by one- and two-stage sintering, to produce low-density (one-stage, 1ST) and high-density (two-stage, 2ST) ceramic samples. Their high-temperature TE properties, namely the electrical conductivity (σ), Seebeck coefficient (α) and power factor (PF), were investigated between 475 and 975 K, in air flow, and related to their respective phase composition, morphology and microstructure. For the 1ST case, the porous samples (56%–61% of ρth) reached maximum PF values of around 210 and 140 μWm^−1^·K^−2^ for the 3% and 6% vol. Co-added samples, respectively, being around two and 1.3 times higher than those of the pure Ca_3_Co_4_O_9_ matrix. Although 2ST sintering resulted in rather dense samples (80% of ρth), the efficiency of the proposed approach, in this case, was limited by the complex phase composition of the corresponding ceramics, impeding the electronic transport and resulting in an electrical performance below that measured for the Ca_3_Co_4_O_9_ matrix (224 μWm^−1^·K^−2^ at 975K).

## 1. Introduction

Thermoelectric (TE) materials can directly convert an applied temperature gradient into electrical voltage due to the Seebeck effect and are regarded as a promising solution for producing electrical power from waste-heat sources [1,2,3]. They are employed in self-sufficient, robust TE devices (modules and generators), which are very reliable, sustainable and scalable, allowing mainly for mobile or remote applications [4,5]. The range of possible applications for TE materials is mostly limited by their relatively low conversion efficiencies [6], but with the recent aid of machine learning and artificial intelligence tools, new horizons in TE materials are envisaged [7]. The performance of a TE material is limited by the Carnot efficiency and is quantified through the dimensionless figure of merit ZT:

(1) $$ZT=\frac{\alpha^{2}\sigma }{\kappa }T$$

combining the absolute Seebeck coefficient (α), electrical conductivity (σ), total thermal conductivity (κ) and prospective working temperature (T). The electrical part of ZT (α^2^σ) is called the power factor (PF) and depends entirely on the material’s intrinsic electrical properties. It becomes obvious from the expression of ZT that good TE materials must simultaneously possess large α and σ and small κ. These TE coefficients, however, are not independent of each other and cannot be treated separately, without affecting the others. For example, the Wiedemann–Franz law addresses the intimate fundamental relationship between the electrical conductivity and the electronic contribution to κ. Therefore, the usual approaches in improving ZT [8] are the decrease of phonon contribution to κ and/or the increase in PF. As most established TE materials are semiconductors, the PF presents a maximum value within a narrow range of charge carrier concentration. The optimization of carrier concentration is usually performed through various electronic band-structure engineering techniques [9,10,11].

Established TE materials like Bi_2_Te_3_, Bi_2_Se_3_, PbTe, half-Heusler alloys, intermetallic Zintl phases, skutterudite and some Si-based alloys have already demonstrated feasible power generation performances (ZT ≈ 1) at low and intermediate temperature ranges [12,13]. At temperatures above ~800–900 K in air, however, they do not possess the necessary thermal and/or chemical stability needed for power generation applications, and they degrade or decompose. Furthermore, they contain expensive, toxic and/or scarce elements which impose important limitations. For these reasons, the established TE materials are usually employed in niche situations where their advantages outweigh their disadvantages [14].

With the discovery of attractive TE properties in Na_x_CoO_2_ ceramics in 1997 [15], a lot of effort has been put in the research and development of CoO-based materials, as well as other transition metal oxides [16], which have important ‘default’ advantages (abundance, low-cost, environmental ‘friendliness’, low reactivity and high thermochemical stability) over established TE materials, enabling them to be considered for power generation applications at high temperatures and in oxidizing conditions [17,18,19,20,21]. While the best performing n-type TE oxides were found in the family of perovskite-type titanates [22,23,24,25], manganites [26,27,28,29] and ZnO-based materials [30,31,32], one of the most promising p-type TE materials (considered as the best choice for a p-type leg in a high-temperature TE module) continues to be the so-called Ca_3_Co_4_O_9_ compound, belonging to the family of misfit-layered cobaltites [26]. However, its main practical drawbacks, namely the strong anisotropic electrical properties induced by the particular misfit crystal structure (which promotes the growth of elongated, randomly oriented plate-like grains of different shapes and sizes, resulting in space-inefficient packing in the bulk ceramics and poor electrical contacts between the grains, under classical sintering/consolidation methods/conditions) and the low bulk density and weak mechanical strength (due to the big difference between the maximum stability temperature of the Ca_3_Co_4_O_9_ phase and the temperature of its liquid phase), represent some of the major challenges in the development of TE modules using Ca_3_Co_4_O_9_ as a p-type leg.

The Ca_3_Co_4_O_9_ compound is an intrinsically nanostructured material that has a monoclinic crystal structure (P2(3) space group) consisting of two different alternating layers, stacked in the *c*-axis direction, namely a distorted triple rock-salt (RS) type Ca_2_CoO_3_ insulating layer, where the cobalt valence is (2+), which is sandwiched between two hexagonal (H) CdI_2_-type CoO_2_ conductive layers (consisting of edge-shared CoO_6_ octahedra), where the mean cobalt valence is between (3+) and (4+). A complex misfit structure is built along the *b*-axis direction [33,34], since the *b* cell parameters are incommensurate (the *a*- and *c*-axes and β angles are common). It is worth mentioning that the CoO_2_ crystallographic planes from Ca_3_Co_4_O_9_ are isostructural to those found in the superconductor Na_0.35_CoO_2_ × 1.3H_2_O, with T_c_ = ~5 K [35]. Based on its crystal structure, Ca_3_Co_4_O_9_ is more technically written with the chemical formula [Ca_2_CoO_3_][CoO_2_]_1.62_, where ‘1.62’ is the so-called incommensurability ratio (*b*_RS_/*b*_H_), found to be responsible for the high Seebeck coefficient values [36], which are susceptible to modifications using chemical substitutions.

Some of the best-known individual or combined approaches for enhancing the TE performances of Ca_3_Co_4_O_9_ include cation substitutions in both calcium and cobalt sites, microstructural engineering techniques and some composite approaches [37,38,39,40,41,42,43,44,45]. The density and grain connectivity may be improved by using specific processing methods, like SPS and LFZ [46,47,48], and high-quality, ultrafine precursor powders [49,50,51].

Two-step sintering schemes also produce quite dense ceramics, but they require long processing/annealing times to stabilize the TE phase [48] and to avoid the formation of unwanted additional secondary phases. According to the equilibrium phase diagram of the Ca-Co-O system in air [52,53], the Ca_3_Co_4_O_9_ phase is stable up to 1199 K, after this limit decomposing to Ca_3_Co_2_O_6_ and CoO, which are both stable up to 1299 K, following the decomposition reactions from the following equations:

(2) $$Ca_{3}Co_{4}O_{9}\;\to \;Ca_{3}Co_{2}O_{6}\;+\;2\;CoO\;+\;½\;O_{2}↑$$

(3) $$Ca_{3}Co_{2}O_{6}\;\to \;3\;CaO\;\left(ss\right)+\;2\;CoO\;\left(ss\right)\;+\;½\;O_{2}↑$$

Following the stoichiometry line further, the first liquid phase appears at 1623 K, a fact which is very important when considering problems related to the bulk density of this phase.

This work aims to study a redox-promoted approach for improving the electrical performance of the bulk Ca_3_Co_4_O_9_ compound, implying an addition of a dispersed metallic phase which further oxidizes on sintering and provides a pore-filling effect. The efficacy of the proposed approach is assessed by measuring the high-temperature TE properties of the resulting composite materials, which are further related to their compositions, morphologies and microstructures.

## 2. Materials and Methods

The Ca_3_Co_4_O_9_ ceramic matrix materials were prepared from ultrafine Ca_3_Co_4_O_9_ precursor powders (batches of ~10 g), obtained through a modified Pechini method. Micrometric metallic Co powder (Alfa Aesar, 1.6 μm, 99.8%, metals basis) was weighed in the stoichiometric amount and dissolved in a minimum amount of medium concentrated nitric acid (HNO_3_, LabKem 65% AGR ISO, ACS) and distilled water. The mixture was slowly heated and magnetically stirred (on a hotplate, with a magnetic stirrer), to evaporate the excess water and to produce the cobalt nitrate, until a relatively viscous pink/violet gel formed. Distilled water and the stoichiometric amount of citric acid monohydrate (Sigma-Aldrich, ACS reagent, ≥99.0%) was added afterward to the mixture, while slowly stirring and heating it continuously to around 423 K, for a minimum of 2 h, and adding distilled water from time to time, to maintain a minimum volume of liquid in the beaker. After this step, the stoichiometric amount of CaCO_3_ powder (Sigma-Aldrich, ACS reagent, chelometric standard, 99.95–100.05% dry basis) was added and left together to mix in the same conditions for ~30 min. Finally, the necessary measured volume of ethylene glycol (Fluka Analytical, puriss. p.a., Reag. Ph. Eur., ≥99.5%) was added to the pink/violet liquid, which started the polymerization reaction, signaled by the appearance of bubbles which later turned to foam, aided by controlling the optimal temperature and stirring values. The liquid mixture from this last part of the modified Pechini chemical synthesis process (which lasted from 3 to 5 h) was subjected to temperatures between 473 and 573 K, on the hotplate; this contributed to the chemical reactions to occur, changing in the last part, after the evaporation of distilled water excess, to a very viscous pink/violet gel or paste, which eventually solidified completely. At this step, the stirring was stopped completely, and the burning of the organic components began, using temperatures between 573 and 673 K, and times between 2 and 4 h. The resulted black powder was finely ground in an agate mortar (aided by high-purity ethanol, which was evaporated afterward) and subjected to a 3-step thermal treatment cycle in air (room temperature (RT) to 573 K (5 K/min), to 873 K (1 K/min, dwell 6 h), to 1073 K (1 K/min, dwell 6 h), and to RT (5 K/min)), in order to decompose the carbonates and burn-out the organic phases excess (controlled combustion), promoting the formation of the desired TE Ca_3_Co_4_O_9_ phase.

The calcined precursor powders were finely ground one last time, in the same way as before, and the various compositions have been produced/prepared, by adding 3%, 6% and 9% vol. of metallic Co and mixing them together, thoroughly. Pure matrix compositions were kept for comparison, as references. The final compositions were uniaxially pressed at 200 MPa (15.7 kN) for around one minute. The green ceramic pellets were subsequently sintered in air, following 2 different sintering schemes inspired from References [49] and [54], to produce low-density and high-density samples, respectively:

One-stage, 1ST: RT to 1173 K (2 K/min, dwell 24 h), to RT (2 K/min).

Two-stage, 2ST: RT to 773 K (8 K/min), to 1473 K (2 K/min, dwell 6 h), to 1173 K (10 K/min, dwell 72 h), to RT (2 K/min).

After sintering, the pellets were carefully polished, finely ground or cut in the adequate shapes and sizes, for the relevant characterization to be performed onward. The experimental densities (ρ_exp_) of Ca_3_Co_4_O_9_-based ceramic samples were determined by geometrical measurements and weighing. The estimated errors in all cases were found to be <3%.

Phase identification was performed through powder X-Ray Diffraction (XRD) analyses, for various Ca_3_Co_4_O_9_-based samples (ground into powder) and for precursors (after the organic phases burn-out and after the 3-step thermal treatment), at RT, using a PHILIPS X’PERT system with CuK_α_ radiation (Cu_α_ = 1.54060 Å), with 2θ angles ranging between 5 and 90 degrees and a step and exposure time of 0.02°2θ and 3 s, respectively.

Morphological characterization of fractured samples coated with carbon was performed using scanning electron microscopy (SEM, Hitachi SU-70 instrument, Aveiro, Portugal), complemented by energy-dispersive spectrometry (EDS, Bruker Quantax 400 detector).

Electrical conductivity and Seebeck coefficient measurements were simultaneously performed on bar-shaped samples (~10 × 2 × 2 mm), in constant air flow, using a custom setup described in detail elsewhere [55], from 475 to 975 K, with a step of 50 K, using a steady-state technique. Freshly cut samples of each composition were fixed inside a specially designed alumina sample holder, placed in turn inside a high-temperature furnace, one horizontally (σ, electrically connected with fine Pt wire, following a four-point probe DC technique arrangement, using an applied electric current) and the other vertically (α, subjected to a local constant temperature difference of ~14 K). The measurement part of the custom setup was similar to that described in Reference [56]. From the measured σ and α values, the PF values were calculated in each case, at each temperature step.

The XRD and TE coefficient plots were constructed using the OrginPro software (2019b (9.65)).

## 3. Results and Discussions

### 3.1. Structural Characterization

The approach proposed in the present work involves the redox-promoted tailoring of the microstructural features, which are known to be of particular importance for the TE performance of Ca_3_Co_4_O_9_. To avoid the formation of excessively porous material with inhomogeneous cations distribution, a chemical synthesis route based on combustion (modified Pechini) was chosen, which provides the necessary high-quality precursor powder, possessing high reactivity, homogeneity and low particle size, leading to desired, single-phase compositions in the case of reference samples [49].

**Figure 1:**
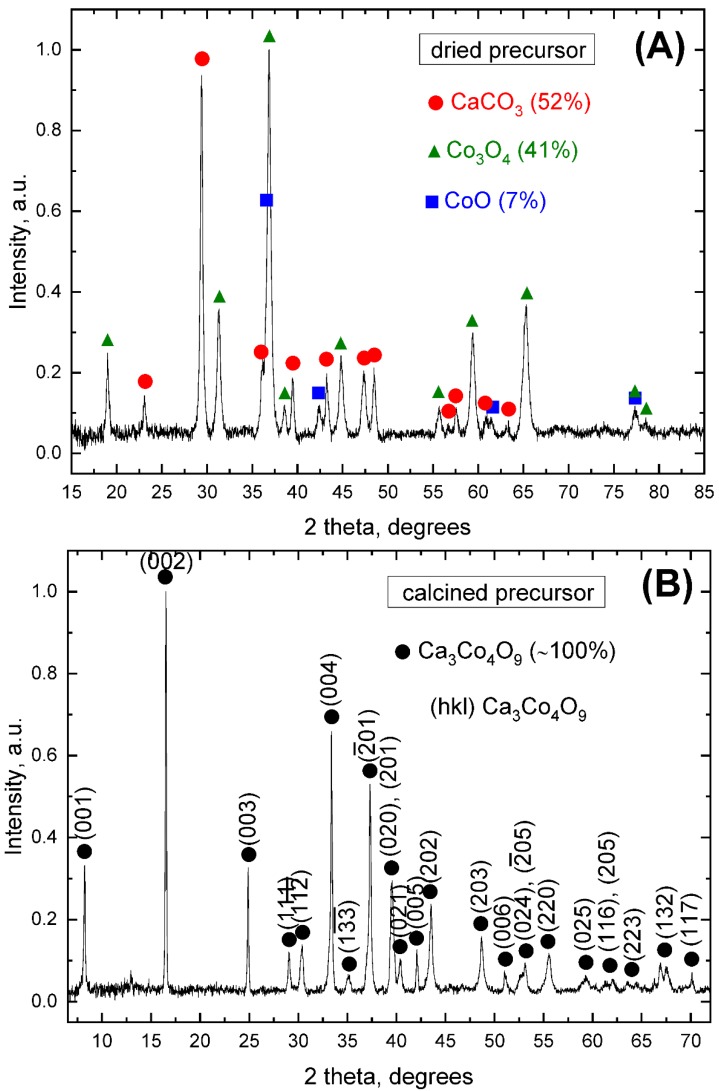
Normalized XRD patterns of the modified Pechini precursor powder: (**A**) after drying at ~623 K for ~3 h (organic phases burn-out) and (**B**) after the three-step thermal treatment at 573 K, 873 K (6 h) and 1023 K (6 h), showing the phase composition and estimated amount in each case. The (hkl) crystallographic planes belonging to the Ca_3_Co_4_O_9_ phase are also shown in (**B**).

From the XRD pattern for dried precursor powder sample, taken immediately after the organic phases combustion/decomposition/burn-out (’dried precursor’, Figure 1A), one can clearly see that the present phases are mainly calcium carbonate and cobalt oxides, in agreement with previously reported results for a similar case [49]. The burn-out temperature (~623 K) from this instance is not high enough to form the desired TE phase.

In agreement with the corresponding equilibrium phase diagram [52,53], the formation of the Ca_3_Co_4_O_9_ phase starts taking place after the application of the three-step annealing cycle, which promotes the following reactions to occur:

(4) $$6CoO+O_{2}\;\to \;2Co_{3}O_{4}$$

(5) $$9CaCO_{3}+4Co_{3}O_{4}+O_{2}\;\to \;3Ca_{3}Co_{4}O_{9}\;+\;9\;CO_{2}↑$$

resulting in nearly single-phase, high-quality precursor powder. The respective XRD pattern can be seen in Figure 1B (‘calcined precursor’), where the corresponding peaks and (hkl) crystallographic planes belong to the Ca_3_Co_4_O_9_ phase, as shown by the PDF cards #00-058-0661 [33] and #00-062-0692.

**Figure 2:**
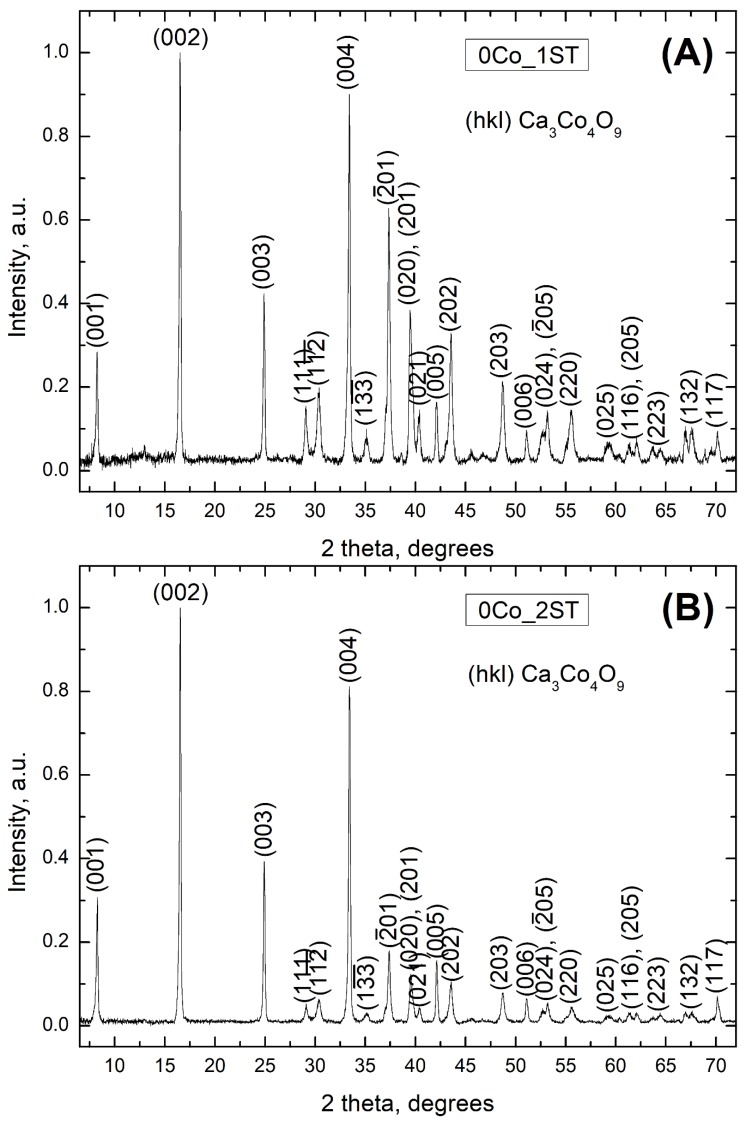

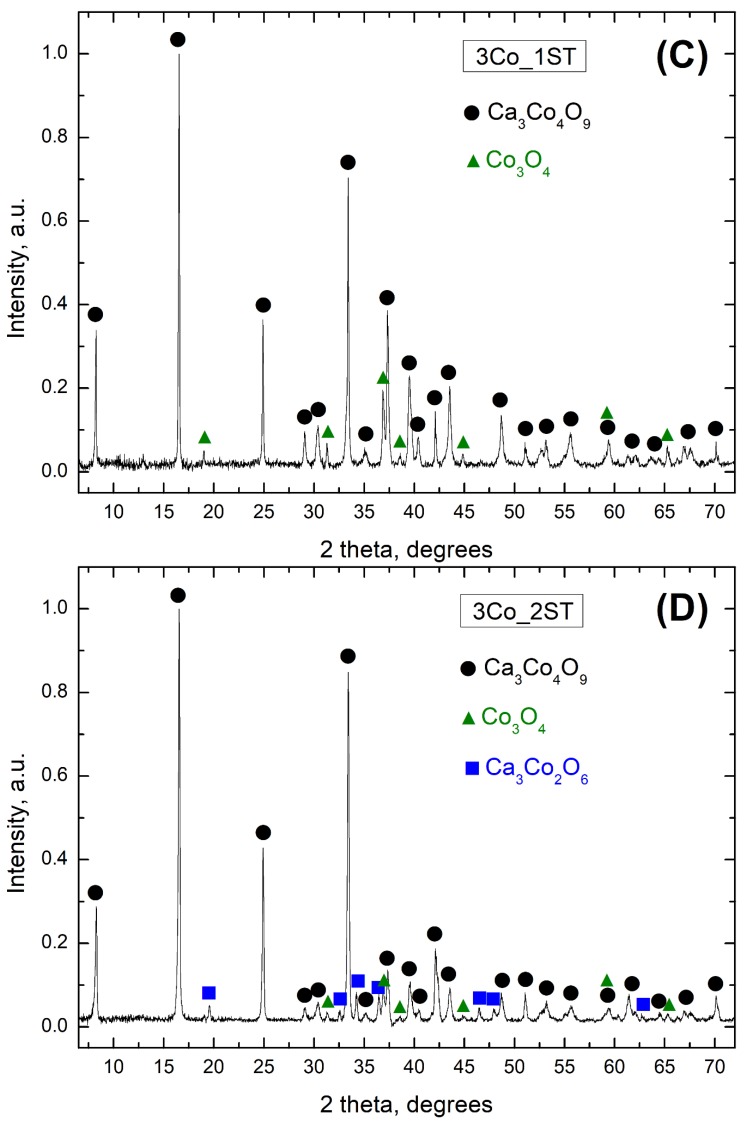

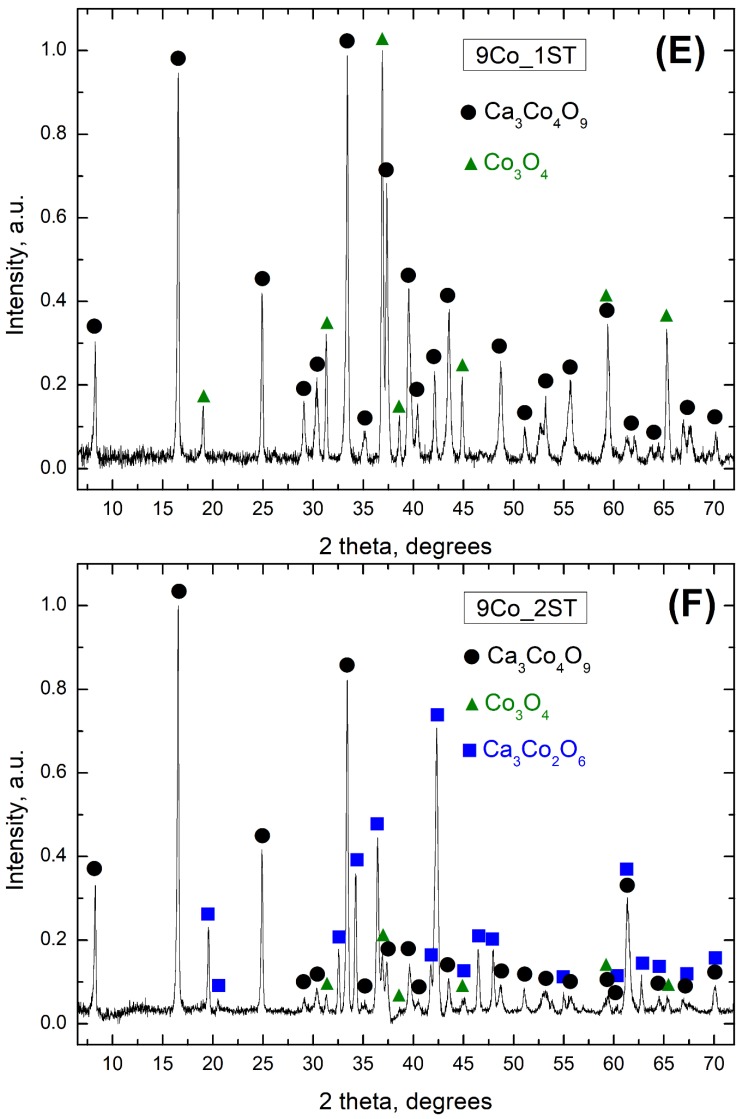
Normalized XRD patterns of the 1ST and 2ST sintered samples: (**A**) 0Co_1ST, (**B**) 0Co_2ST, (**C**) 3Co_1ST, (**D**) 3Co_2ST, (**E**) 9Co_1ST and (**F**) 9Co_2ST, showing the phase composition in each case. In (**A**,**B**), the (hkl) crystallographic planes belonging to the Ca_3_Co_4_O_9_ phase are shown.

Going forward to the 1ST and 2ST sintered samples obtained from the modified Pechini precursor powder, the XRD results (Figure 2A–F) clearly show the presence of additional factors affecting the final phase composition in all cases, being more simple in the 1ST case and more complex in the 2ST case, as compared to the pure matrix samples. From now on, the 0%, 3%, 6% and 9% vol. Co containing Ca_3_Co_4_O_9_ samples sintered in one and two stages will be denoted as 0, 3, 6 or 9Co_1ST or 2ST, for simplicity, as shown in Table 1. Table 1 also shows the fractions of Ca_3_Co_4_O_9_ phase and phase impurities in wt.%, as estimated by the RIR method.

Firstly, the XRD patterns corresponding to both pure matrix compositions 0Co_1ST and 0Co_2ST (Figure 2A,B) indicate the presence of single-phase Ca_3_Co_4_O_9_, as marked by the corresponding (hkl) crystal planes, in agreement with the work of Masset et al. [33] and other literature references [52,53]. Thus, in terms of the phase composition, there is no significant difference between the two sintering schemes applied to the pure matrix samples; these samples are further used as references to follow the effects imposed by the cobalt additions. The XRD data for the Co-containing samples (3, 6 and 9Co_1ST and 2ST) suggest the presence of new phases, different for each sintering scheme, as shown in Figure 2C–F. Those secondary phases correspond to Co_3_O_4_ for the 1ST case, and Co_3_O_4_ and Ca_3_Co_2_O_6_ for the 2ST sintered samples, respectively. Their concentration, estimated using the RIR method, increases with the addition of metallic Co (Table 1).

Metallic cobalt readily oxidizes in the air, at temperatures above 900 K, forming a CoO and Co_3_O_4_ mixture [57]. Hence, the observed phase composition is in good agreement with that predicted by the phase diagrams [52,53]. Shifting from the nominal 3CaO–2Co_2_O_3_ ratio to the Co-rich region mainly promotes the formation of Ca_3_Co_4_O_9_ + Co_3_O_4_ at temperatures below 1060–1200 K (1ST case), while the formation of Ca_3_Co_2_O_6_ and Co(Ca)O takes place at higher temperatures (2ST case). The re-equilibration of the Ca_3_Co_2_O_6_ and Co(Ca)O mixture at 1173 K during the second step from the 2ST sintering is only partial, explaining why the resulting composites from this case still retain a significant fraction of Ca_3_Co_2_O_6_ phase.

### 3.2. Microstructural Evolution

Some additional insights on the observed difference in phase composition between the 1ST and 2ST sintered samples can be obtained by analyzing the densities of the prepared ceramic materials (Table 1). The relative density of the 1ST samples was calculated by assuming a simple mixing rule and the theoretical densities of 4.69 and 6.06 g/cm^3^ for Ca_3_Co_4_O_9_ and Co_3_O_4_, correspondingly [58]. The density shows a noticeable improvement for the 3Co_1ST samples, as compared to the pure matrix; the values for 6Co_1ST and 9Co_1ST are also slightly higher than for the reference, but slightly lower than for 3Co_1ST. Together with the electron microscopy results discussed below, these results suggest that the addition of metallic cobalt particles contributes by filling the undue porosity during oxidation. This porosity is formed by the plate-like grains of different shapes and sizes and by the low packing density resulting from the 1ST sintering scheme. At high cobalt contents, this effect vanishes, probably due to the excessive expansion provided by the cobalt oxide formation and corresponding detachment of the particles/grains. It should be noticed that the 9Co_1ST samples also show a relatively poor mechanical strength, as compared to the other 1ST samples. In any case, the apparently low-density values of the 1ST sintered samples are typical for this material, obtained in this manner [59].

On the other hand, rather dense samples were obtained in the 2ST sintering case. The relative density was not assessed for these samples due to possible errors originating from a more complex phase composition. Nevertheless, the tendency for a general density increase as compared to the pure matrix composition is evident, the corresponding ρ_exp_ being comparable with those found in the literature, e.g., for microwave processed samples [40]. Although simultaneously resulting in a more complex phase composition, the relatively high processing temperature actually facilitates the densification of the samples. At the same time, the first temperature step at 1473 K significantly suppresses the oxygen exchange/diffusion in the samples and delays the phase composition equilibration during the second step, 1173 K, in the window where the TE Ca_3_Co_4_O_9_ phase is formed. Thus, the final composition of the 2ST sintered samples correspond to a kinetically frozen state, representing a significant fraction of high-temperature Ca_3_Co_2_O_6_ phase. Similar observations have been reported elsewhere [48].

The significant porosity of the 1ST sintered samples, known to be detrimental for the electrical performance, actually facilitates the thermal equilibration, due to a faster oxygen diffusion, which results in the formation of a higher amount of Ca_3_Co_4_O_9_ phase for the same nominal composition, as compared to the equivalent 2ST case.

**Figure 3:**
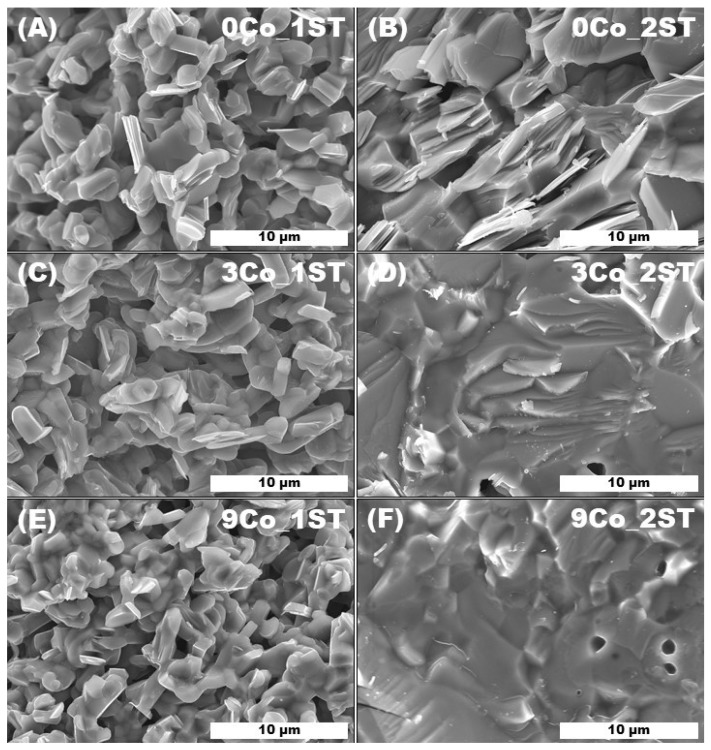
Representative SEM micrographs of fractured 1ST and 2ST sintered samples: (**A**) 0Co_1ST, (**B**) 0Co_2ST, (**C**) 3Co_1ST, (**D**) 3Co_2ST, (**E**) 9Co_1ST and (**F**) 9Co_2ST.

**Figure 4:**
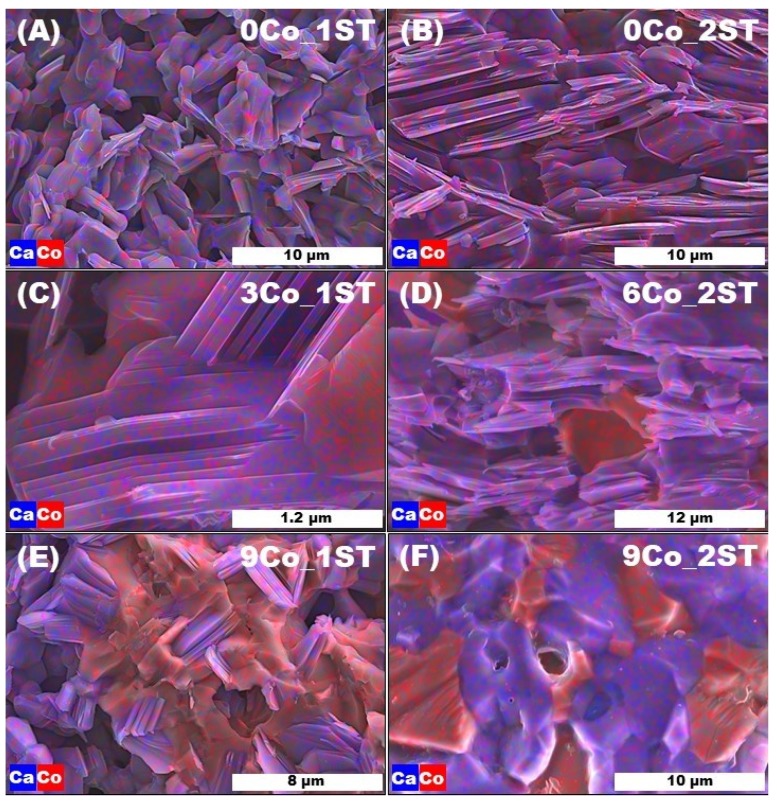
Representative EDS maps of fractured 1ST and 2ST sintered samples: (**A**) 0Co_1ST, (**B**) 0Co_2ST, (**C**) 3Co_1ST, (**D**) 6Co_2ST, (**E**) 9Co_1ST and (**F**) 9Co_2ST.

The above discussion is in good agreement with the following microstructural results (Figure 3 and Figure 4). For the 1ST case, the representative SEM images from fractured samples shown in Figure 3A,C,E apparently suggest an improvement in densification from 0Co_1ST to 3Co_1ST, while the morphology of the grains remains essentially unchanged. The high porosity found for the 9Co_1ST sample is also visible in the micrograph E (Figure 3). The small grain sizes and low particle-size dispersion, typical for the Pechini precursor powders, can also be seen in these selected SEM pictures from the 1ST sintered samples, where the mean grain sizes are estimated to be ~1 µm.

For the 2ST case, however, the morphologies and microstructures of the samples change substantially, especially for the Co-containing ones (Figure 3B,D,F). First of all, the porosity is much lower than in the 1ST case, promoted by the additional high-temperature sintering step, which, together with the long annealing time, also led to a twofold or threefold increase in grain size (Figure 3B) and a better consolidation during sintering. Furthermore, when Co is added to the Ca_3_Co_4_O_9_ matrix, the plate-like grains associated to the Ca_3_Co_4_O_9_ phase become increasingly harder to identify in the micrographs of the corresponding samples, due to the increase in secondary phases amount (especially Ca_3_Co_2_O_6_), in agreement with the corresponding XRD results shown previously.

The respective representative EDS maps from Figure 4 show more details on the phase evolution and explain the different morphologies and microstructures found for the two sintering schemes employed. Firstly, the EDS maps from Figure 4A,B suggest a uniform Ca and Co distribution in the 0Co_1ST and 0CO_2ST samples, in agreement with their pure phase composition (Figure 2A,B and Table 1), showing 100% Ca_3_Co_4_O_9_. The microstructural arrangements observed for 3Co_1ST and, especially, 9Co_1ST (Figure 4C,E) indicate that Co_3_O_4_, which forms on oxidation of Co particles simultaneously with the formation of Ca_3_Co_4_O_9_, may actually connect highly asymmetric Ca_3_Co_4_O_9_ grains between themselves, acting as a pore filler in the 1ST samples with low-density grain packing. These connections are fewer and more homogeneously distributed for 3Co_1ST, while their number increases and their homogeneity decreases (agglomerates start to form gradually) on increasing the amount of added cobalt. The large agglomerates of Co_3_O_4_ from 9Co_1ST may explain the higher porosity and subsequent lower density of these samples.

The effect of improved grain connection vanishes in the 2ST sintered samples, where high density is inherent due to processing at a higher temperature. In this case, the excess of cobalt leads to rather complex morphologies and microstructural features, determined by an interplay between distinct formation kinetics of Ca_3_Co_4_O_9_, Ca_3_Co_2_O_6_ and Co_3_O_4_, affecting their spatial distribution. In fact, the grain morphology of these phases becomes essentially similar for 9Co_2ST (Figure 4F), in contrast to 9Co_1ST (Figure 4E), where elongated Ca_3_Co_4_O_9_ grains are preserved and interconnected by Co_3_O_4_ inclusions.

The results presented up to this point are in agreement with the following high-temperature electrical performances’ results.

### 3.3. Electrical Performance

**Figure 5:**
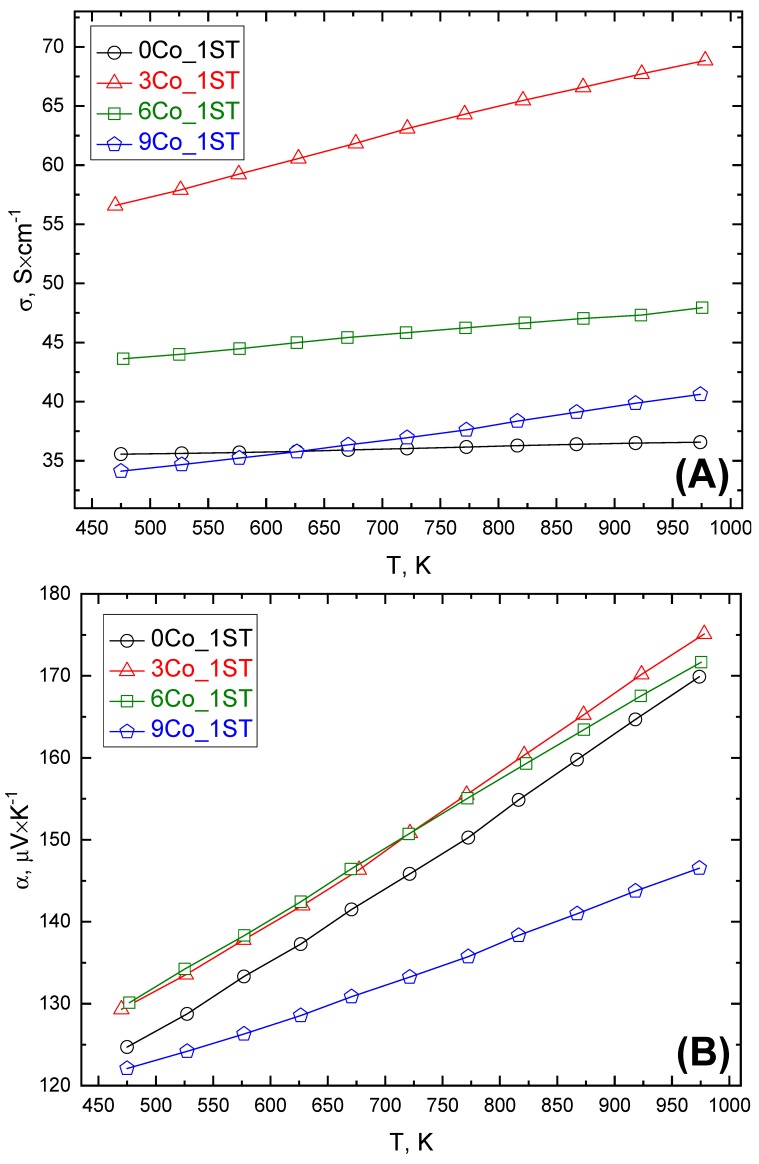

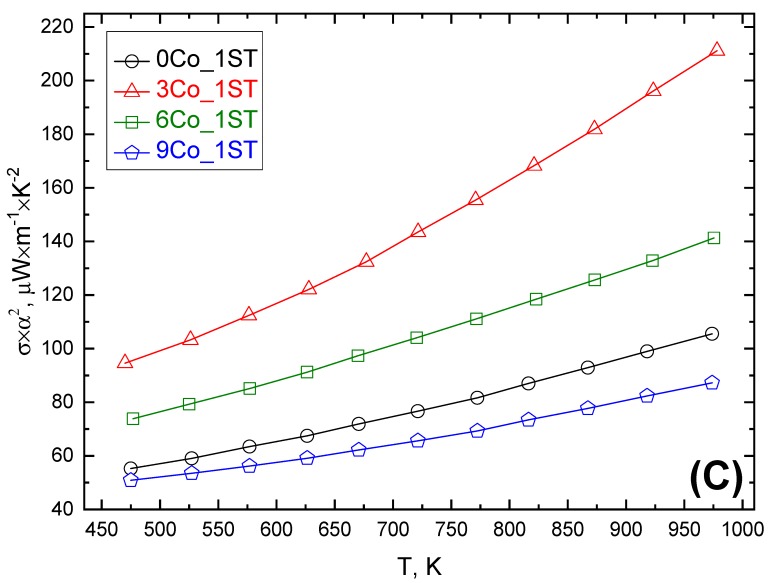
Electrical conductivity (**A**), Seebeck coefficient (**B**) and power factor (**C**) for 1ST sintered samples.

The evolution of the electrical conductivity (*σ*) with temperature for the 1ST sintered samples is shown in Figure 5A. All *σ* values increased almost linearly with temperature, showing a typical semiconducting behavior (*dσ*/*dT* ≥ 0), also found elsewhere in literature, for similar cases [45,50,54,60,61]. The high-temperature electrical conduction mechanism characteristic for Ca_3_Co_4_O_9_ is thermally activated hole hoping [62]. The electrical conductivity of Ca_3_Co_4_O_9_ is highly anisotropic [13] and is mainly governed by the holes from the *ab* plane [33], i.e., by the conductive hexagonal [CoO_2_] layers. The formation of elongated grains (with mostly random orientation) during conventional solid-state processing results in high porosity and poor grain interconnectivity.

The obtained results unambiguously suggest that the proposed redox tailoring approach indeed results in a significant improvement of the charge carrier transport in the 1ST sintered materials. The electrical conductivity for the sample with the lowest amount of added cobalt, 3Co_1ST, is 1.6–1.9 times higher than for the pure matrix reference. It should also be noted that the highest *σ* value of 68 Scm^−1^, observed for 3Co_1ST at 975 K, is higher than some of the best-reported values in the literature [54,63]. This enhancement is believed to result from the improved grains interconnectivity, provided by the oxidation of added cobalt particles and filling of the pores with cobalt oxide. An oxidation-promoted filling by Co_3_O_4_ expansion in the pores is essential in this case, while direct sintering of Ca_3_Co_4_O_9_ and Co_3_O_4_ mixture produces only a marginal improvement of the electrical conductivity in SPS processed samples, as found by F. Delorme [45]. It should be noticed that the electrical conductivity of 3Co_1ST with 61% of relative density (Table 1) is only slightly (~10% to 15%) lower than for 98% dense Ca_3_Co_4_O_9_ prepared by SPS [45]. Even though Co_3_O_4_ intrinsically possesses notably lower total conductivity than Ca_3_Co_4_O_9_ [45,64] and, thus, cannot provide a decisive enhancement of the charge carrier concentration, it contributes, however, by improving the charge carrier mobility, by connecting neighboring Ca_3_Co_4_O_9_ grains, following the following basic equation [12]:

(6) σ=neμ

where *n, e* and *μ* are the charge carrier concentration, the electron charge and the carrier mobility, respectively. This hypothesis is also confirmed by comparable values of the Seebeck coefficient for 0Co_1ST, 3Co_1ST and 6Co_1ST; for the latter two composites, the thermopower is even slightly higher than for the pure matrix reference. This difference also agrees with the fact that the Seebeck coefficient of Co_3_O_4_ is noticeably higher compared to Ca_3_Co_4_O_9_ [65], but appears counterintuitive when considering the significant drop in *α* observed for the 9Co_1ST sample (Figure 5B). A similar decrease was actually observed by F. Delorme [45] and attributed to the presence of the compressive strain originating from the mismatch of the thermal expansion coefficients of Ca_3_Co_4_O_9_ and Co_3_O_4_, which is more likely to contribute at relatively higher Co_3_O_4_ contents, as those assessed in Reference [45]. The highest *α* values were measured for both 3 and 6Co_1ST, reaching the maximum value of 175 μVK^−1^ at 975 K for 3Co_1ST, which is comparable to some of the best values reported in the literature [45,46].

The resulting PF values for the 1ST case are shown in Figure 5C. All samples demonstrate similar behavior: the PF values increase proportionally with temperature, in the whole measured temperature range. The PF values systematically decreased from 3Co_1ST to 9Co_1ST, following the corresponding trends observed for electrical conductivity and Seebeck coefficient. The highest PF value of 210 μWm^−1^·K^−2^, belonging to 3Co_1ST at 975 K, is among the best values reported in the literature, for highly textured, high-density samples [45,46].

**Figure 6:**
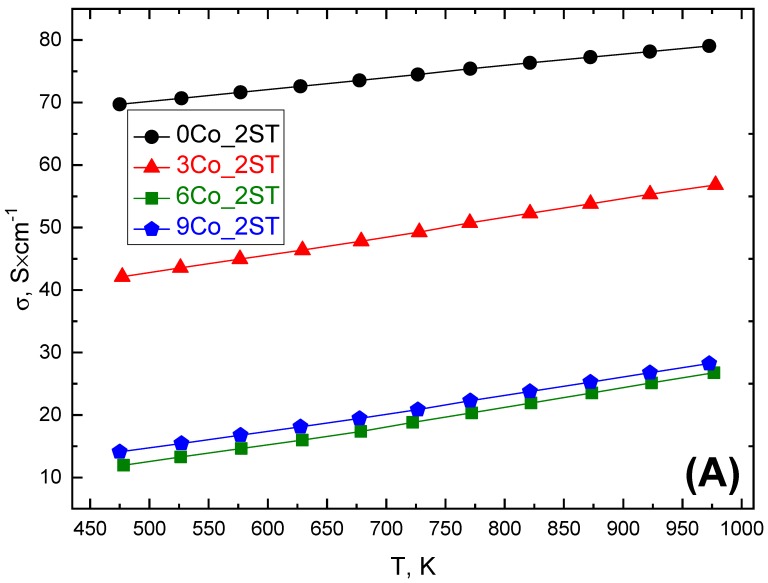

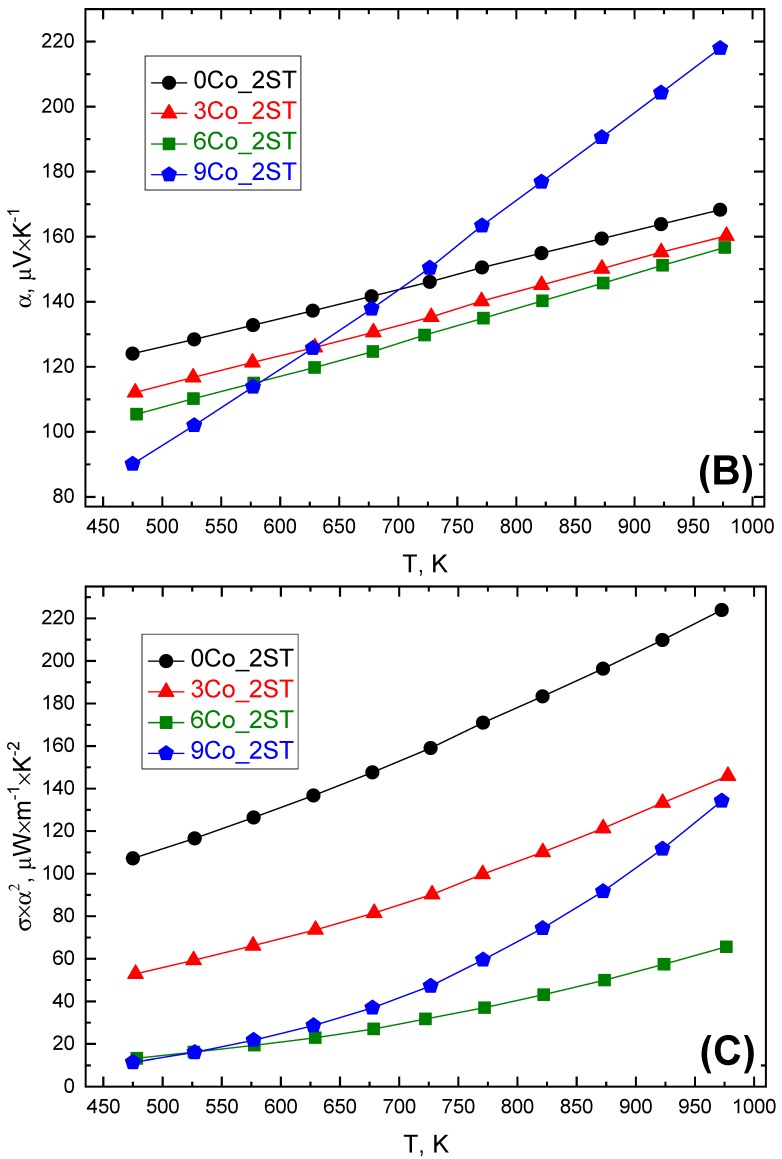
Electrical conductivity (**A**), Seebeck coefficient (**B**) and power factor (**C**) for 2ST sintered samples.

Essentially opposite effects of the cobalt addition on the electrical transport properties can be observed for the denser 2ST sintered samples (Figure 6). In this case, all *σ* values also increased linearly with temperature, in the whole measured temperature range, with the highest conductivity values measured for the reference 0Co_2ST sample. The lower *σ* values measured for the cobalt-added samples can most probably be explained by the presence of the additional secondary phase, Ca_3_Co_2_O_6_, besides Co_3_O_4_, known to possess *σ* values of at least one order of magnitude lower than for Ca_3_Co_4_O_9_ [66,67]. The lowest *σ* values (between 13 and 28 Scm^−1^) measured for 6 and 9Co_2ST are in agreement with the largest 23 and 40 wt.% fraction of Ca_3_Co_2_O_6_, respectively, found in these samples. The reference sample 0Co_2ST has the largest *σ* values (between 70 and 80 Scm^−1^) measured in this work, due to its higher density (80% of ρ_th_); this conductivity range is, again, comparable to some of the best-reported values from the literature [54,63]. In 3Co_2ST and 6Co_2ST, the cobalt addition also surprisingly resulted in a general decrease of the Seebeck coefficient (Figure 6B), which might be a result of various mechanical strains imposed by the complex phase composition. The combination of these factors resulted in a general decrease of the power factor measured for all cobalt-added 2ST sintered samples, as shown in Figure 6C.

**Figure 7:**
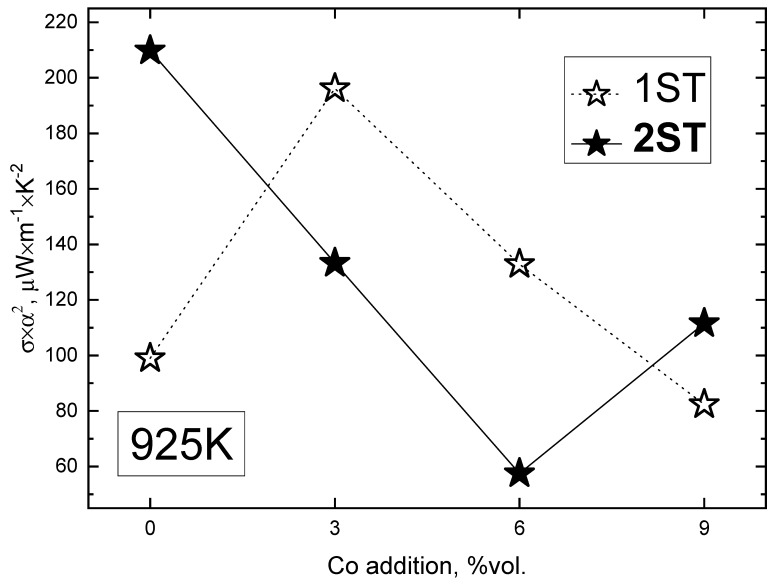
Compositional dependence of the power factor at 925 K, for the 1ST and 2ST sintered samples.

Finally, Figure 7 illustrates the compositional dependence of electrical performance at a fixed temperature.

The highest PF values (around 200 μWm^−1^·K^−2^) from this work were achieved for 0Co_2ST and 3Co_1ST, due to the high density and grains interconnectivity improvement effect provided by the oxidation of cobalt, respectively. Higher cobalt additions promoted the formation of a larger amount of resistive secondary phases, which lead to lower PF values in the corresponding samples. Since a significant improvement of the electrical performance was found for a relatively low Co concentration, additional studies in the range of 1–5% vol. of cobalt addition might be necessary to further evaluate the potential of the proposed approach. The kinetics of pore-filling effects can be also adjusted by the size of the cobalt particles and the heating/dwell conditions. Thus, the scope of the present work includes the first demonstration of the redox-tailoring effects provided by the metal powder addition on the electrical counterpart of the TE performance of Ca_3_Co_4_O_9_-based materials. Further assessment of the impact of the redox-induced interface on the thermal transport properties in such composite materials is also essential and needs to be addressed.

## 4. Conclusions

This work demonstrates a new processing route for tailoring the high-temperature electrical performance of Ca_3_Co_4_O_9_-based materials, based on redox-promoted pore-filling effect on oxidation of metallic cobalt particles added to the Ca_3_Co_4_O_9_ matrix. The efficacy of the proposed approach was shown to be strongly dependent on the processing conditions, resulting in a significant enhancement of the thermoelectric performance for relatively porous materials, where the effect of pore filling was more pronounced. This was provided by the presence of Co_3_O_4_ phase, which promoted an improvement in densification and better interconnectivity between Ca_3_Co_4_O_9_ grains, simultaneously resulting in enhanced electrical transport. An alternative two-stage sintering route led to much denser Ca_3_Co_4_O_9_/Ca_3_Co_2_O_6_/Co_3_O_4_ ceramic samples, possessing, however, lower electrical performances due complex phase composition. The highest PF value of 210 μWm^−1^·K^−2^ at 975 K was observed for the Ca_3_Co_4_O_9_-based ceramics containing 3% vol. of metallic cobalt addition, which is among the best-reported values in the literature for textured, high-density p-type layered cobaltites. The results highlight the importance of a proper grain boundaries design in Ca_3_Co_4_O_9_-based thermoelectrics, where inherently poor densification during normal sintering can be compensated by introducing a suitable grain-connecting phase.

## Figures and Tables

**Table 1:** Denominations, phase composition and density of the prepared ceramic samples.

Composition	Processing Conditions	Denomination	Phase Composition, wt.%*	Density ρ_exp_, g/cm^3^	ρ_exp_/ρ_th_**
Ca_3_Co_4_O_9_	one-stage	0Co_1ST	Ca_3_Co_4_O_9_(100)	2.62	0.56
Ca_3_Co_4_O_9_ +3% vol. Co	one-stage	3Co_1ST	Ca_3_Co_4_O_9_(94); Co_3_O_4_(6)	2.90	0.61
Ca_3_Co_4_O_9_ + 6% vol. Co	one-stage	6Co_1ST	Ca_3_Co_4_O_9_(85); Co_3_O_4_(15)	2.85	0.59
Ca_3_Co_4_O_9_ + 9% vol. Co	one-stage	9Co_1ST	Ca_3_Co_4_O_9_(80); Co_3_O_4_(20)	2.81	0.57
Ca_3_Co_4_O_9_	two-stage	0Co_2ST	Ca_3_Co_4_O_9_(100)	3.74	0.80
Ca_3_Co_4_O_9_ + 3% vol. Co	two-stage	3Co_2ST	Ca_3_Co_4_O_9_(94); Ca_3_Co_2_O_6_(4); Co_3_O_4_(2)	4.12	-
Ca_3_Co_4_O_9_ + 6% vol. Co	two-stage	6Co_2ST	Ca_3_Co_4_O_9_(70); Ca_3_Co_2_O_6_(23); Co_3_O_4_(7)	4.35	-
Ca_3_Co_4_O_9_ + 9% vol. Co	two-stage	9Co_2ST	Ca_3_Co_4_O_9_(40); Ca_3_Co_2_O_6_(40); Co_3_O_4_(20)	4.49	-

*—Estimated using the RIR method; **—Theoretical density.
